# The Advancing Mental Health Equality Collaborative: Using Quality Improvement to Advance Equality in Mental Health Care – CORRIGENDUM

**DOI:** 10.1192/bjo.2023.605

**Published:** 2023-10-23

**Authors:** Adele de Bono, Matt Milarski, Renata Souza, Saiqa Akhtar, Rosana Bevan, Emily Cannon, Tom Ayers, Viviana Aya, Laura-Louise Arundell, Mark Farmer, Meera Burgess, Lade Smith, Rajesh Mohan, Amar Shah

This article was originally published with a co-author's name spelled incorrectly. The error has been corrected and the online PDF and HTML versions updated.
